# Reduced Progression of Cardiac Allograft Vasculopathy with Routine Use of Induction Therapy with Basiliximab

**DOI:** 10.5935/abc.20150063

**Published:** 2015-08

**Authors:** Ricardo Wang, Lidia Ana Zytynski Moura, Sergio Veiga Lopes, Francisco Diniz Affonso da Costa, Newton Fernando Stadler Souza Filho, Tiago Luiz Fernandes, Natália Boing Salvatti, José Rocha Faria Neto

**Affiliations:** 1 Santa Casa de Curitiba, Curitiba, PR - Brazil; 2 Pontifícia Universidade Católica do Paraná, Curitiba, PR - Brazil

**Keywords:** Vascular Diseases / physiopathology, Heart Transplantation, Antibodies, Monoclonal, Murine-Derived / admininstration & dosage, Immunosuppressive Agents

## Abstract

**Background:**

Cardiac allograft vasculopathy (CAV) is a major limitation for long-term survival of patients undergoing heart transplantation (HT). Some immunosuppressants can reduce the risk of CAV.

**Objectives:**

The primary objective was to evaluate the variation in the volumetric growth of the intimal layer measured by intracoronary ultrasound (IVUS) after 1 year in patients who received basiliximab compared with that in a control group.

**Methods:**

Thirteen patients treated at a single center between 2007 and 2009 were analyzed retrospectively. Evaluations were performed with IVUS, measuring the volume of a coronary segment within the first 30 days and 1 year after HT. Vasculopathy was characterized by the volume of the intima of the vessel.

**Results:**

Thirteen patients included (7 in the basiliximab group and 6 in the control group). On IVUS assessment, the control group was found to have greater vessel volume (120–185.43 mm^3^ vs. 127.77–131.32 mm3; p = 0.051). Intimal layer growth (i.e., CAV) was also higher in the control group (27.30–49.15 mm^3^ [∆80%] vs. 20.23–26.69 mm^3^ [∆33%]; p = 0.015). Univariate regression analysis revealed that plaque volume and prior atherosclerosis of the donor were not related to intima growth (r = 0.15, p = 0.96), whereas positive remodeling was directly proportional to the volumetric growth of the intima (r = 0.85, p < 0.001).

**Conclusion:**

Routine induction therapy with basiliximab was associated with reduced growth of the intima of the vessel during the first year after HT.

## Introduction

With increased survival among heart transplantation (HT) patients, mainly due to improvements in immunosuppression, the incidence of late complications, including cardiac allograft vasculopathy (CAV)^1^, has increased. CAV is characterized by progressive obliteration of vessels due to intimal proliferation and is considered a major cause of graft dysfunction in the first year after HT and the second most common cause of long-term death^2^.

Lymphocytes play an important role in both acute and chronic graft rejection. The immunological and non-immunological factors implicated in the pathogenesis of CAV converge by activating T lymphocytes (TL)^3^, as demonstrated by Nagano et al.^4^. Animal models in which these cells were blocked did not develop vasculopathy^5^. Thus, T lymphocyte blockade has been the objective of therapies for the prevention of CAV^6^.

Basiliximab is a chimeric antibody receptor antagonist of interleukin 2 (IL-2) and is indicated in induction therapy for patients at high risk of rejection after organ transplantation^7^. IL-2 is a potent immunomodulator that plays an important role in the activation and maintenance of the immune response and lymphocyte proliferation^8^; furthermore, it is a key step in the development of acute rejection^9^. Blockage of TL proliferation and reduced acute rejection can delay the onset of CAV^10^. The aim of this study was to determine whether blockage of IL-2 with basiliximab early in the transplantation process has an effect superior to placebo in decreasing the growth of the vessel intima during the first year following HT.

## Methods

We conducted a retrospective analysis of the database from a single center, including patients who underwent HT from September 2007 through March 2009. The patients were separated in two groups according to the induction therapy: those treated with basiliximab (Simulect®; Novartis, NJ, USA) and those who received no induction therapy (control group). In our institution the use of basiliximab became routine in July 2008; therefore, a comparison was made to a series of cases before and after this period. In this period, there was no difference regarding surgical technique, preservation, or other adjuvant medications. We included only patients who had clinical and ultrasound follow-up for at least 1 year. We excluded patients who did not comply with intravascular ultrasound (IVUS) follow-up or whose images in the database were inadequate to allow such analysis. The study was approved by local Ethics Committee (protocol 0005154/11).

### Endpoints

The primary objective was to compare the two groups with regard to volumetric growth of the intimal layer measured by IVUS after 1 year. The secondary objective was to evaluate the remodeling of the vessel and lumen volume and donor atherosclerosis.

### Immunosuppression protocol

Immunosuppression was performed in the basiliximab group at a dose of 20 mg IV, together with 500 mg methylprednisolone in three daily doses and 150 mg mycophenolate mofetil (MMF) in two doses on the day of transplantation; on the fifth day, another dose of 20 mg IV basiliximab was administered; on that day, therapy with cyclosporine was initiated. In the control group, immunosuppression was conducted with methylprednisolone and MMF at the same dosage; in addition, cyclosporine was initiated on the day of transplantation at the same dosage.

### Evaluation of vasculopathy

As part of the HT protocol, patients are routinely evaluated with angiography and intracoronary ultrasound (IVUS) only at the left anterior descending (LAD) artery. This evaluation is performed 30 days after HT and then repeated annually.

Coronary angiography and IUVS were performed concurrently with an endomyocardial biopsy. To perform the procedure, a 6F introducer was introduced into the femoral artery, followed by catheterization of the left coronary artery. Unfractionated heparin (100 IU/kg) was instilled intravenously together with an intracoronary dose of isosorbide mononitrate (10 mg). The ultrasound examination was performed with an Atlantis® catheter (Boston Scientific Scimed Inc., Maple Grove, Minn.) and a 4.3 Fr catheter with a 40-MHz transducer. The IVUS catheter was positioned in the distal LAD artery; automatic pullback was performed with a velocity of 1 mm/s and an acquisition rate of 30 frames/s. The images were stored on a compact disk and analyzed using ILab® software (Boston Scientific Scimed, Inc.).

### IVUS Analysis

To provide monitoring of the same segment, a 10-mm segment was selected just after the output of the first diagonal. Segment analysis was methodologically validated in a manner similar to that previously described^11,12^. Analysis was performed on the first computed tomography (CT) slice after the departure of the diagonal branch, marking the beginning of the segment; then each image is evaluated every 30 cuts (1-mm interval between analyses), until 10 segment images (10 mm) are completed. The analysis consists of a manual outlining of the lumen and external elastic membrane (EEM), calculating the lumen area and EEM area. Measurements were performed as standardized by the American College of Cardiology/European Society of Cardiology^13^. The intimal area was calculated by subtracting the area of the lumen minus EEM. Calculation of the volume of the vessel lumen and intima was carried out using the method described by Simpson^11^. The volume percent was calculated according the following formula: {∑ (EEM area − lumen area)/∑ EEem area × 100.

### Statistical Analysis

Continuous data were expressed as median plus 25^th^ and 75^th^ percentiles. Categorical data were expressed as absolute numbers. Nonparametric tests were used to evaluate differences in continuous data, and due to the small sample size, we used Mann–Whitney test for evaluation of the differences in IVUS findings. A simple linear regression model was used to assess the relationship between previous atherosclerosis and intimal growth as well as the relationship between intimal and vessel growth after 1 year, using Pearson correlation coefficients. For categorical data, the differences were evaluated using Fisher's exact test. A two sided p-value < 0.05 was required for statistical significance. Analyses were performed with SPSS 12.0 software (Chicago, IL, USA).

## Results

In the period from 2007–2009, 23 HTs were performed in our institution. Two patients died during the perioperative period, and three during the first year of follow-up. Two patients were excluded from the present study due to inadequate IVUS images, and 3 patients only underwent IVUS study beyond 13 months of follow-up. We evaluated 13 patients, of whom 7 received basiliximab (basiliximab group) and 6 did not (control group). Demographic data are listed in Table 1. The patients were predominantly male (n = 10); the median age was 55 years in the basiliximab group and 47.5 years in the control group. Three patients in the control group developed acute renal failure in the postoperative period, characterized by a serum creatinine > 0.5 mg/dL, whereas no patients in the basiliximab group developed this complication. The levels of total cholesterol, triglycerides, and angiotensin receptors were similar between the groups (p = NS), and creatinine levels were somewhat higher in the control group. The use of inhibitors and statins was higher in the basiliximab group. Only a few patients received everolimus/sirolimus during follow-up: one in the basiliximab group and two in the control group. However, all patients received mycophenolate mofetil. No patient received a diagnosis of cytomegalovirus confirmed by serology. The number of rejection episodes was similar in both groups. Two patients in the basiliximab group and three in the control group underwent a biopsy with 2R; they required hospitalization and underwent pulse therapy with intravenous corticosteroids.

**Table 1:** Patient demographics

	Basiliximab group (n = 7)	Control group (n = 6)	p value
Male sex (n)	4	6	N.S. *
Age (years)	55 [40-65]	47.5 [40-59]	N.S.
Diabetes mellitus	3	2	N.S.
Renal failure after transplantation	0	3	N.S.
Rejection	6	6	N.S.
**Biopsy (during first year after transplantation)**			
0R	2	1	N.S.
1R	3	2	N.S.
2R	2	3	N.S.
**Coronary angiography**			
ISHLT CAV_0_†	7	6	N.S.
Total cholesterol (mg/dL)	229 [179-243]	180[152-249]	N.S.
Triglycerides (mg/dL)	223 [176-450]	150 [129.2-232]	N.S.
HDL (md/dL)	48 [36-52]	38 [28-44]	N.S.
Glucose	85 [83-98]	93 [82-105]	N.S.
Creatinine	1.2 [1.2-1,4]	1.6 [1.4;-1.6]	N.S.
Imunossupressor:			N.S.
Prednisone	6	5	N.S.
Micophenolate mofetil	7	6	N.S.
Cyclosporine	6	5	N.S.
Everolimus/rapamicin	1	2	N.S.
**Other medications**			
Statin	4	1	N.S.
Angiotensin converting enzyme inhibitor	6	4	N.S.
Insulin	0	1	N.S.

* N.S.: Not significant;

† ISHLT CAV_0_: International Society of Heart Lung Transplantation definition of cardiac allograft vasculopathy (reference: JHLT 2010;29(7):717-727.)

**Graph 1:**
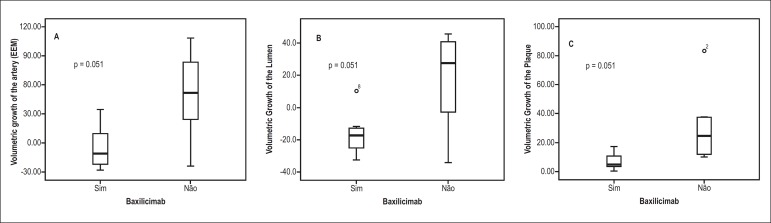
Analysis of the volume change at 1 year after transplantation. A: Variation in the vessel. B: Variation in the lumen. C: Variation in the intima

**Graph 2:**
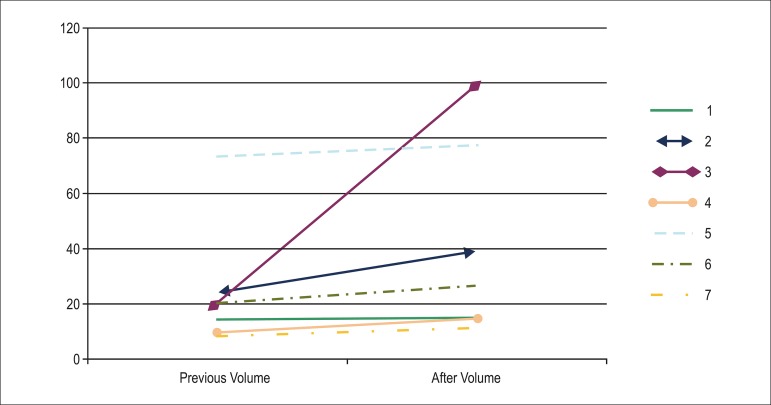
Growth of plaque volume in patients undergoing induction therapy with basiliximab

**Graph 3:**
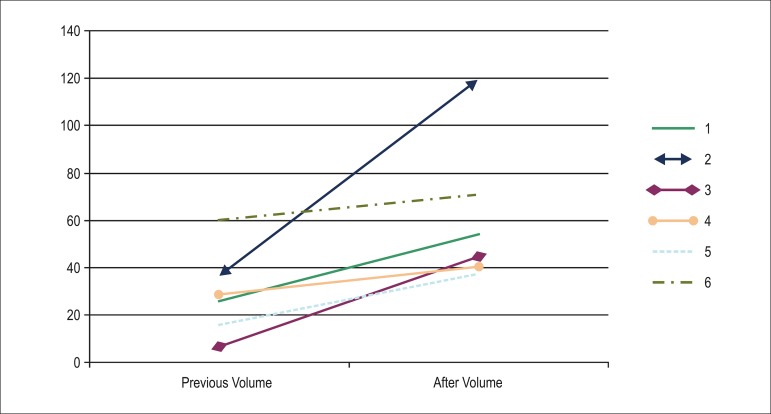
Plaque volume in patients who received no induction therapy

Coronary angiography performed during the first year following HT did not detect the presence of significant vascular disease (e.g., CAV), based on the new classification of the International Society of Heart and Lung Transplantation (ISHLT)^14^. The data obtained by IVUS are presented in Table 2 and Graph 1. In the control group, vessel volume (delineated by the EEM) exhibited positive remodeling (increase in volume growth of 49.39 mm^3^), whereas in the basiliximab group, the effect was reversed (negative remodeling: –4.17 mm^3^), with a trend toward statistical significance (p = 0.051). The findings were similar with regard to luminal volume (-11.53 × 17.3 mm^3^; p = 0.051). Regarding the intimal layer (plate), a higher rate of growth (follow-up volume minus baseline volume) occurred in the control group (baseline value: 27.3 mm^3^; control group: 49.15 mm^3^; basiliximab group: 20.23–26.69 mm^3^; p = 0.015; Graphs 2 and 3).

**Table 2:** Analysis of volumes obtained with IVUS

	Vessel Previous	Vessel after	**Lumen previous**	Lumen after	Intima previous	Intima after	
Basiliximab group	131.32 [101.69;202,06]	127.77 [110.39;174,05]	113.22 [82.67;144,70]	99.23 [87.86;123,36]	20.23 [9.65;29,11]		26.69 [14.65;39,24]
Control group	120.77 [111.92;191,57]	185.43 [142.23;229,76]	103.31 [86.52;149,16]	134.96 [105.50;158,79]	27.30 [13.65;42,41]		4915 [39.76;82,89]
p value	1,00	0,042	1,00	0,05	0,62		0,05

**Graph 4:**
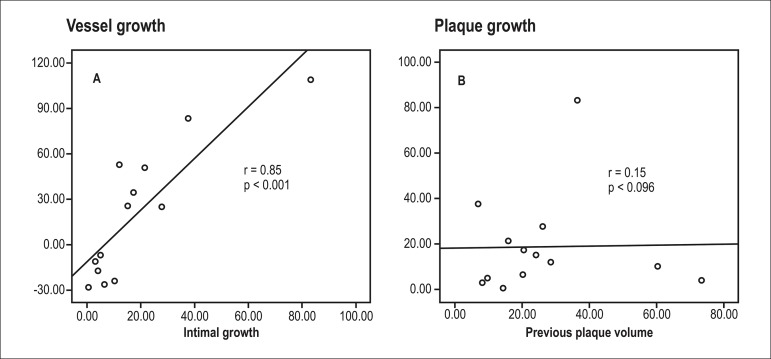
A. Vessel growth according to intimal growth. B. Impact of previous plaque atherosclerosis on CAV growth

In simple linear regression analysis assessment (Graph 4B), previous atherosclerosis was not associated with increased growth of the intima (r = 0.15; p = 0.96). Positive remodeling (increase in EEM) was associated with a greater increase in intimal volume (r = 0.85; p < 0.001; Graph 4A).

## Discussion

This study revealed the following findings. (1) The use of induction therapy with basiliximab was associated with less intimal tissue growth in the first year after HT. (2) In the control group, we observed greater positive remodeling, which was probably related to increased intimal growth observed in this group. (3) With simple linear regression analysis, vessel growth was proportional to the increase of the plaque regardless of induction therapy. (4) Atherosclerosis in the donor was not associated with increased growth of the intima.

Graft vascular disease begins with endothelial injury, followed by a repair process, cell proliferation, and accumulation of extracellular matrix^3,6^. The degree of organ preservation, ischemia/reperfusion injury, acute rejection, and viral infection (particularly cytomegalovirus) are cited as the main non-immunological factors that affect the endothelium in the first year after HT. In response to injury, endothelial cells express cell adhesion molecules (vascular cell adhesion molecule, intercellular cell adhesion molecule, and selectins); furthermore, recruitment of inflammatory cells and release of proinflammatory cytokines occur. This results in a vicious cycle of chronic inflammation, culminating in the obliteration of the lumen^15-17^.

Growth inhibition by basiliximab, which exhibits its action 4–6 weeks after infusion^9^, reinforces the relationship between early recruitment of lymphocytes and the appearance of CAV^6^. Tori et al.^18^ and Young et al.^19^ observed that the infiltration and activation of lymphocytes in the first days after HT are already sufficient for the appearance of CAV. The specific activation pathway of major histocompatibility complex II and proliferation of Th1 lymphocytes are considered to be the primary route of CAV formation^20^. Blocking various parts of this pathway has been proven effective in reducing the appearance of CAV^21^. IL-2 also plays a major role in the activation pathway of T helper 1 (Th1) lymphocytes, and this could explain the benefit of the use of basiliximab in the first weeks after HT to interrupt the cycle of injury and repair, thus preventing the chronic inflammatory process.

The reduction of intimal growth induction therapy is not a new finding^22^. Zhang et al.^22^ observed that induction therapy with antithymocyte antibody (antithymocyte globulin, ATG) delays the onset of CAV. However, the effect did not translate into increased long-term survival. In addition, a higher incidence of cancer is observed in patients treated with ATG, which may explain the higher late mortality rate in this group. Long-term follow-up is indicated to determine the benefit and/or clinical harm of this therapy. As basiliximab is not associated with increased infection or neoplasia^9,23^, we expect a clinical benefit.

In the global registry of the ISHLT, the use of basiliximab for induction therapy has a neutral effect on CAV (relative risk [RR]: 1.16; confidence interval [CI]: 0.99–1.37); however, CAV increased with the use of muromonab-CD3 (OKT3; RR: 1.17; p = 0.038)^2^. This effect is probably due to selection bias. Patients with a higher risk of acute rejection in the post-transplantation period and those who have higher levels of a reactor panel of antibodies (PRA) are at greatest risk of developing CAV^24,25^. Another example of selection bias occurs with induction therapy, correlates with IL-2 receptor antagonists, and a risk of renal dysfunction, and this medication is indicated for patients at high risk for renal failure after transplantation^26^.

As in atherosclerosis^27^, we observed positive remodeling to accommodate the increase of the intima, thus avoiding involvement of the arterial lumen. In previous studies, most intimal tissue growth and positive remodeling occurred during the first year post-HT^28,29^. From the second year onward, despite a lower growth of the intima, there is greater involvement of the arterial lumen due to negative vessel remodeling^28^. We found variation in the natural history of the process in patients treated with basiliximab. We also found a slight decrease in vessel remodeling and luminal volume reduction; however, to date, we do not know how it will progress following the second year.

In our institution, induction therapy with basiliximab is routinely performed with the goal of delaying the onset of the need for caucineurin inhibitors and minimizing the nephrotoxic effects of cyclosporin^26,30^. Candidates for HT have a high prevalence of renal dysfunction; furthermore, after HT, renal function may deteriorate, particularly because of the use of nephrotoxic drugs, low cardiac output, and impaired cardiopulmonary bypass. Moreover, acute renal failure is associated with a poor outcome^2^.

Due to low sensitivity of coronary angiography in detecting early CAV, IVUS is used in our institution for CAV research, because its high sensitivity and specificity provide an earlier diagnosis of CAV^14,31^. Clinically, IVUS has a good correlation with angiography; thus, it is a good prognostic tool^32^. Some evidence exists that early diagnosis of CAV, together with the adjustment of immunosuppressive therapy is associated with growth control. Furthermore, some studies have reported regression of CAV^21,33,34^. The volumetric measurement of the plate by IVUS has been previously validated by experimental^11^ and clinical studies^12^. This methodology has a strong correlation with histomorphometry. Moreover, it is a robust method and requires a smaller sample to demonstrate the effectiveness of strategies that have an impact on reducing the intima^11^.

The major limitation of this study is its small sample size, possible bias in patient selection, and retrospective nature. Thus, a prospective, multicenter, randomized study with a larger sample size, which extends clinical follow-up to assess the long-term benefit, is indicated. Furthermore, our control group had greater plaque volume, probably due to atherosclerosis of the donor; this may have affected the outcome, as suggested by a recent study by Yamasaki et al.^35^. However, in our study, plaque volume did not correlate with higher growth of the intima (r = 024; p = 0.94), a finding that is consistent with those of previous studies^36,37^.

## Conclusion

In this retrospective analysis, induction therapy with basiliximab was associated with less volumetric growth of intimal tissue (graft vasculopathy) in the first year after HT.
